# Perceptions Toward an Attentional Bias Modification Mobile Game Among Individuals With Low Socioeconomic Status Who Smoke: Qualitative Study

**DOI:** 10.2196/59515

**Published:** 2025-05-09

**Authors:** Michael Wakeman, Lydia Tesfaye, Gunnar Baskin, Tim Gregory, Greg Gruse, Erin Leahy, Brandon Kendrick, Sherine El-Toukhy

**Affiliations:** 1 Division of Intramural Research, National Institute on Minority Health and Health Disparities, National Institutes of Health, 11545 Rockville Pike, Rockville, MD, 20852, United States, 1 3015944743; 2 ICF, Reston, VA, United States

**Keywords:** serious games, focus groups, smoking cessation, attentional bias modification, mobile interventions, young adults

## Abstract

**Background:**

Attentional bias modification (ABM) can help address implicit attention from individuals who smoke toward smoking cues, which trigger cravings and lapses that impede smoking cessation. We developed an ABM mobile game, Fruit Squish, to support individuals who smoke and are quitting as part of a multicomponent smoking cessation mobile app, Quit Journey. Users advance in the game by tapping on neutral (ie, fruit) rather than smoking-related (eg, cigarette pack) imagery that they are presented with, essentially training them to avoid focusing on smoking cues.

**Objective:**

This study aimed to gauge acceptance of an ABM smoking cues mobile game among young adults who smoked and were socioeconomically disadvantaged.

**Methods:**

We recruited 38 individuals who smoked cigarettes, aged 18‐29 years, who were neither 4-year college graduates nor enrollees in 4-year colleges to participate in 12 semistructured digital focus groups. Sessions were audio recorded and transcribed verbatim. We used ATLAS.ti software to code the transcripts for salient themes based on the Second Unified Theory of Acceptance and Use of Technology constructs (ie, effort expectancy, facilitating conditions, hedonic motivation, performance expectancy, and social influence) and sentiment (ie, negative, neutral, and positive).

**Results:**

Performance expectancy of the mobile game was the dominant technology acceptance construct discussed (34/110, 30.90%). Perceived usefulness of the game was mixed in sentiment owing to perceptions that the game aimed to distract individuals who smoke during cravings and concerns that cue imagery in the game could trigger cravings. Hedonic motivation was the second most discussed technology acceptance construct (17/110, 15.45%), with participants describing the game as neither fun nor engaging. Participants referenced their past experiences with mobile games and mobile device characteristics as facilitating conditions for using the game (10/110, 9.09%). Although effort expectancy was minimally discussed (6/110, 5.45%), the game was characterized as easy to use. To improve the game, participants suggested adding new levels with increasing difficulty (eg, increase stimuli speed and limit session time) and new game elements (eg, leaderboard). Other suggestions included improving game graphics and renaming the game to capture its relation to smoking cessation.

**Conclusions:**

Young adults with low socioeconomic status who smoke had mixed reactions to a mobile smoking cues ABM game. Results suggest the need to communicate the rationale underlying ABM games to users and their potential positive effects on smoking cessation. To promote the uptake and sustained use of ABM mobile games, they need to be on par with commercially available entertainment mobile apps. Research is needed to explore the efficacy of gamified ABM on cognitive biases in real-life settings.

## Introduction

Implicit cognitive processes play a critical role in the maintenance of addictive behaviors, including tobacco use disorders [1,2]. Individuals who smoke have unconsciously enhanced attention toward smoking cues, which are stimuli associated with smoking behaviors ranging from physical objects to smells and observed behaviors [3-5]. Attentional biases result from positive rewards associated with smoking that are conditioned over time. Accordingly, attentional bias toward smoking cues can trigger cravings and have negative affect, which in turn cause smoking behaviors or lapses [6]. Furthermore, attentional biases can persist among individuals who formerly smoked and are associated with a heightened risk of relapse [7-9]. Attentional biases have been documented through both self-reports (eg, self-reported cravings after exposure to cues) and objective measures (eg, faster reaction times when responding to smoking cues) [8,10]. Several theories explain the development of attentional biases, including the incentive-sensitization theory, the elaborative intrusion theory of desire, and the theory of current concerns [1,2]. Collectively, these models suggest that substance cues do not merely trigger cravings but that the two exist as part of a bidirectional relationship and positive feedback cycle [1,2].

Clinical guidelines for treating nicotine dependency recommend that individuals who smoke avoid or reduce exposure to smoking cues in their environments [11]. Individuals who smoke can follow these recommendations for smoking cues present in their immediate environments (eg, ashtrays in their homes). However, these cues can be pervasive and beyond their control in other instances (eg, media and advertisements) and are, thus, more difficult to avoid [5,12]. This is particularly true for disadvantaged individuals who smoke, are exposed to a high volume of tobacco advertising, are targets of tobacco marketing tactics (eg, coupons), and reside in areas with a higher density of tobacco retailers [13-15]. For instance, retail marketing (eg, tobacco price promotions) disproportionately targets African Americans [16]. Similarly, tobacco retailer density is higher in African American, Hispanic, and lower-income neighborhoods [15]. These strategies contribute to the saliency of tobacco-related imagery, which triggers smoking behaviors and lapses, and are a potential mechanism for higher smoking prevalence and difficulty quitting among populations at the receiving end of these strategies [17].

Cognitive bias modifications (CBMs) are interventions that target and restructure implicit cognitive biases related to disorders, such as substance use, using varied cognitive tasks [18]. Attentional bias modification (ABM) is a CBM intervention approach where individuals are trained to refocus their attention away from substance cues, thereby severing previously formed associations [2]. One approach involves a visual-probe task where both neutral and substance-related stimuli are presented on a screen followed by a probe (eg, a small dot) to redirect the user’s attention to the neutral stimuli [19]. ABM has previously been explored for addictive behaviors, including tobacco and alcohol use, with evidence of their effects on substance use being mixed. For example, a review of meta-analyses found that ABM can be effective in shifting attentional biases, but the effect sizes were short-lived and generally modest for appetitive stimuli (eg, smoking) [18]. Another meta-analysis focusing on tobacco and alcohol found that CBM has a small reductive effect on cognitive biases but did not reduce substance use [20]. These results could be attributed to several factors, mainly the limited number of training sessions [18]. However, there is evidence that multiple (vs single) ABM sessions can decrease attentional bias over longer time spans [21,22].

Smartphones, with their high use, market penetration, and advanced features [23], can conveniently deliver multisession, self-administered ABM interventions to individuals who smoke in their natural environments. More importantly, advancements in mobile technologies afford opportunities to gamify ABM interventions to engage target populations and increase their adherence to ABM interventions over multiple sessions. The use of mobile apps to deliver CBM for substance use disorders (eg, alcohol use) has been promising [24,25]. For example, a review of trials using apps to deliver CBM interventions found that 7 of 8 trials effectively modified existing biases or reduced related symptoms (eg, anxiety and alcohol consumption). Nearly all of these apps relied on the visual-probe task [26]. In the context of smoking cessation, few attempts have been made to translate ABM techniques outside laboratory settings using digital technologies and platforms (eg, personal digital assistants and web-based interventions) [2]. Further, a review identified only 3 commercially available CBM apps for tobacco use (ie, Quitty, StopSmoking-Quit Smoking, Quit Smoking: Simple and Quick), none of which were supported by published literature [26]. Although limited in number, studies examining smoking-related ABM delivered via smartphones have primarily used modified visual-probe tasks and had mixed effects on attentional biases and no effects on symptoms (eg, cravings and smoking behaviors) [27-29].

**Figure 1.**
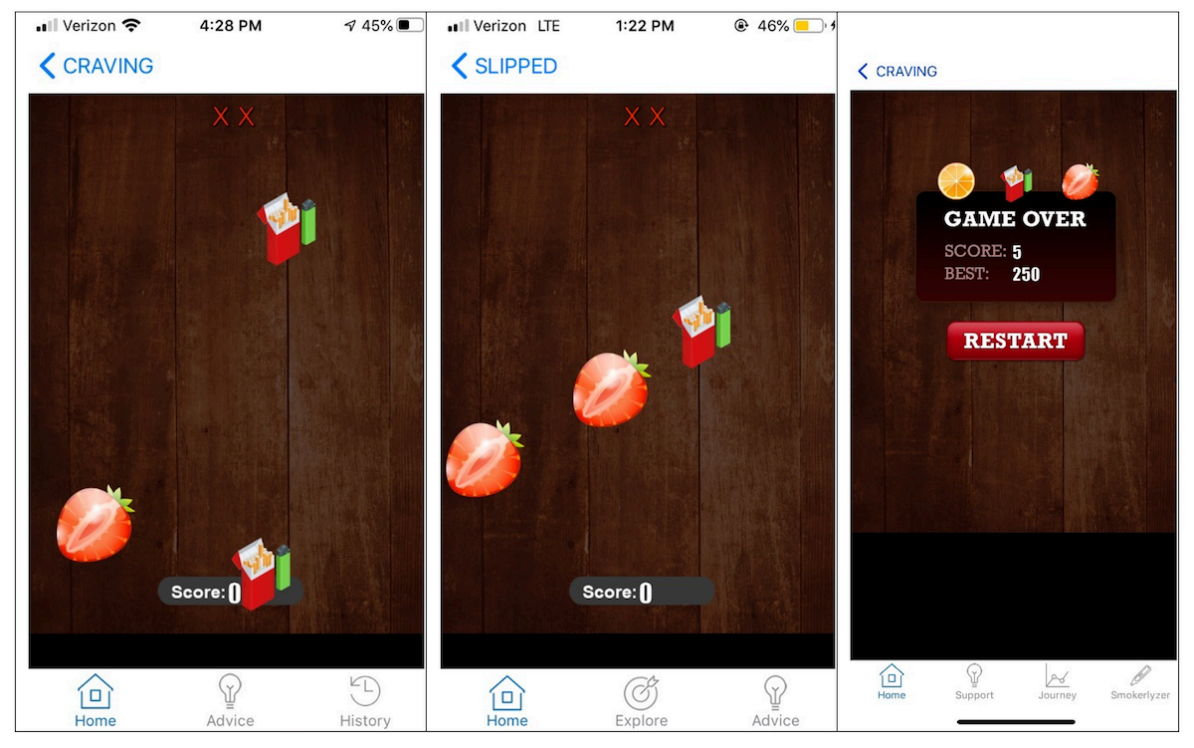
Screenshots from Fruit Squish.

To our knowledge, gamifying mobile ABM interventions has been limited to substance use disorders (ie, alcohol and opioid use) primarily using modified visual-probe tasks. Gamified ABM interventions have yet to be implemented in smoking cessation apps as part of multicomponent interventions [30]. To that effect, we developed a smoking cues ABM mobile game called Fruit Squish. The game aims to help individuals who smoke reduce or avoid attention paid to smoking cues, a behavioral change technique that facilitates smoking cessation [31,32]. Briefly, the game mimics popular mobile games (eg, Fruit Ninja) where players must slice select stimuli (eg, pieces of fruit) rather than undesired stimuli (eg, bombs) to advance. We translated the visual-probe task typically used in attentional bias training to a Fruit Ninja game template by substituting images of bombs with smoking-related images (eg, cigarette packs). In Fruit Squish, players must tap on the fruit (rather than smoking-related images) to receive points and advance in the game (Figure 1). The game is part of Quit Journey, a novel smoking cessation mobile app targeting individuals with low socioeconomic status who smoke. Briefly, Quit Journey is a multicomponent smoking cessation intervention that includes features common to smoking cessation apps (eg, educational content). Additionally, the app includes 4 novel features: carbon monoxide physiological tracking, performance-based coupon incentives, an ABM game (ie, Fruit Squish), and a user-generated message library with smoking-related messages (feature under development) that provides access to on-demand messages and to just-in-time support messages that are sent when app users are at risk of smoking [33, Tesfaye et al, 2024; Wakeman et al, 2024, unpublished data]. Due to the novelty of ABM games in the context of smoking cessation, understanding users’ perceptions of them is important. Prior research has established that user perceptions of new technologies are essential for their acceptance and use [34,35]. In this study, we gauge perceptions of individuals with low socioeconomic status who smoke toward a smoking cues ABM mobile game, Fruit Squish, to further improve the game as a feature of Quit Journey. The study falls under the preparation phase of the multiphase optimization strategy for developing and evaluating behavioral interventions [36,37].

## Methods

### Ethical Considerations

The National Institutes of Health Institutional Review Board deemed the study exempt on October 11, 2019. ICF’s Institutional Review Board deemed the study exempt on November 19, 2019 and approved an amendment on February 26, 2020. All participants received information about the study and its procedures and verbally consented to participate in the study before the audio recording began. Userworks, Inc (Silver Spring, MD), a user experience and usability research firm, had access to personally identifiable information for participant recruitment and screening purposes, whereas all authors did not. UserWorks assigned enrolled participants identification numbers (eg, P1), which were used during the focus group discussions, and subsequently in the transcripts and data analysis. Participants received US $150 gift cards as an incentive.

### Participants and Recruitment

We partnered with Userworks to recruit a convenience sample of 38 participants through its research panels and other commercial platforms (eg, Craigslist). UserWorks contacted candidate participants via email and screened those interested over the phone for enrollment eligibility (Note S1 in Multimedia Appendix 1). Participants were eligible for inclusion in the study if they self-reported being 18‐29 years old, neither 4-year college educated nor enrolled in a 4-year college as an indicator of low socioeconomic status [38-41], current cigarette smokers who smoked at least 100 cigarettes in their lifetime and smoked every day or some days, willing to quit smoking within 6 months, not using smoking cessation aids, not using noncigarette combustible tobacco products (eg, cigars), smartphone owners, and able to speak English. Recruitment occurred between January and April 2020 and no participants withdrew after enrollment. Recruitment was not based on data saturation. However, the sample size was deemed sufficient to achieve data saturation [42]. The 32-item consolidated criteria for reporting qualitative research checklist appears in Table S1 in Multimedia Appendix 1 [43].

### Procedures

We held 12 semistructured focus groups to explore perceptions of a newly developed smoking cessation mobile intervention, Quit Journey, and its features, including the ABM mobile game described here, among young adults who smoked and were socioeconomically disadvantaged. The game was presented as a concept in 8 predesign focus group sessions and as a prototype version in 4 postdesign sessions. A total of 12 participants were involved in both pre and postdesign focus group sessions. Discussions lasted ≈1.5 hours and were held on GoTo Meeting, audio recorded, and transcribed verbatim. Transcripts were autogenerated by GoTo Meeting, verified by 3 members of our team, and were not returned to participants for comment.

Discussions followed a moderation guide based on the technology acceptance models (Note S2 in Multimedia Appendix 1) [34,35]. TG, a user experience strategist and researcher (male), was the primary moderator, while EL, a strategic communications and marketing project director (female), was the backup moderator and note-taker. TG had training and certification in user research, including focus groups. The moderators had no prior relationship with participants who were informed that the moderators were unaffiliated with the research group that commissioned the study. Only the participants, TG, EL, and SEL, were present during the focus group sessions. Before conducting the focus groups, we conducted dry runs.

### Analysis

Following a deductive thematic analysis [44,45], we developed a codebook and corresponding themes using 5 constructs from the Second Unified Theory of Acceptance and Use of Technology: effort expectancy, facilitating conditions, hedonic motivation, performance expectancy, and social influence [34,35]. These constructs were defined as follows: (1) effort expectancy referred to "perceived ease of use or effortfulness with which one can navigate mobile applications and their features and seamlessly integrate them in one's life"; (2) facilitating conditions were defined as "factors that can aid or impede the uptake or use of mobile applications or their features. These include individual-related (eg, skills, predispositions, prior experiences) and technical-related (eg, infrastructure) factors". (3) Hedonic motivation referred to "perceived fun, pleasure, or enjoyment (or lack thereof) associated with the use of mobile applications and their features". (4) Performance expectancy referred to "perceived usefulness or helpfulness of smoking cessation mobile applications and their features in achieving desired health goals and behaviors". (5) Social influence referred to "perceived importance of significant others' recommendations and approval of using mobile applications and their features" [33,46,47, Wakeman M et al, 2024; Tesfaye L et al, 2024, unpublished data]. Quotes that did not fit under a technology acceptance construct were coded as "not applicable". Additionally, we included sentiment (ie, negative, neutral, and positive), design concepts, and users’ suggestions. Positive and negative sentiment captured statements that indicated “a sense of approval, praise, or certainty” or “a sense of disapproval, criticism, or skepticism about any aspect of smoking cessation mobile applications and their features such as their worthiness, utility and impact, time and effort investment, and compatibility with one's life,” respectively. Neutral sentiment captured statements "that were neither positive or negative in tone, contained an equal number of positive and negative remarks, or were conditional (ie, positive in nature but depended on the presence or absence of another factor" [33,46,47, Tesfaye et al, 2024; Wakeman et al, 2024, unpublished data]. The design concepts code captured statements related to "user interface, content input and delivery, information structure, navigation, typography, and aesthetics of smoking cessation applications or their features" [33,46,47, Tesfaye et al, 2024; Wakeman et al, 2024, unpublished data]. Finally, the suggestions code captured statements that were “concerned with improvements, modifications, or additions to smoking cessation applications or their features aimed to improve their functionality or design" [33,46,47, Tesfaye et al, 2024; Wakeman et al, 2024, unpublished data]. We created additional codes for emerging themes (ie, intent/willingness to use the game, novelty). We coded the transcripts for salient themes applying only one theme from each semantic domain to a quote. When multiple themes were present, we selected the predominant or underpinning theme in conditional quotes (eg, a quote was coded as hedonic motivation if the participant considered the game to be useful if it was fun and engaging). We coded the transcripts for all Quit Journey app features, but only present here results related to the smoking cues mobile game. Our research on the use of mobile technologies for smoking cessation and on Quit Journey and its features has been published elsewhere or is currently under review [33,46,47, Tesfaye et al, 2024; Wakeman et al, 2024, unpublished data].

A member of our team extracted quotes for exploratory analysis using Excel (Microsoft Corporation). Two authors (MW and LT) independently coded focus group transcripts using ATLAS.ti (version 8; ATLAS.ti Scientific Software Development GmbH), which was also used to calculate intercoder agreement [48]. We calculated the Krippendorff c-α, a measure of separating relevant and irrelevant content whereby coders code texts of similar locations and lengths, and the Krippendorff cu-α, a measure of semantic domain reliability whereby coders code a given text for the presence or absence of a semantic domain [48]. Discrepancies were resolved through discussions between MW, LT, and SEL. Qualitative results were not returned to participants for feedback.

## Results

### Patient Characteristics

The Krippendorff c-α was 0.82, whereas the Krippendorff cu-α was 0.66 for technology acceptance, 0.70 for sentiment, and 0.94 for Quit Journey app features. Sample characteristics appear in Table 1, and detailed participant characteristics in Table 2.

**Table 1.** Participant characteristics (N=38) [33,46,47].^a^

Characteristic	Values, n (%)
Sex	
Female	20 (52.63)
Male	18 (47.36)
Race and ethnicity	
NH^b^ American Indian or Alaska Native	1 (2.63)
NH Asian, Native Hawaiian or Pacific Islander	3 (7.89)
NH Black or African American	11 (28.94)
Hispanic or Latino	6 (15.78)
NH White	16 (42.10)
Mixed	1 (2.63)
Highest level of education	
Less than high school	3 (7.89)
High school graduate	10 (26.31)
High school equivalent	3 (7.89)
Some college, no degree	18 (47.36)
2-year associate degree	4 (10.52)
Smoking frequency	
Every day	30 (78.94)
Some days	8 (21.05)
Quit timeframe	
7 days	11 (28.94)
30 days	22 (57.89)
6 months	5 (13.15)
Smartphone operating system	
Android	21 (55.26)
iOS	17 (44.73)

a Includes unpublished data from Tesfaye et al, 2024; Wakeman et al, 2024.

b NH: Non-Hispanic.

**Table 2.** Detailed participant characteristics [33,46,47].^a^ Participants who identified as Hispanics or Latinos were considered as such regardless of race.

ID	Sex	Race and ethnicity	Highest level of education	Smoking frequency	Quit timeframe	Smartphone operating system
P01^b^	Female	NH^c^ White	Some college, ND^d^	Every day	30 days	Android
P02^b^	Male	Hispanic/Latino	Some college, ND	Every day	30 days	Android
P03	Female	Hispanic/Latino	HS^e^ incomplete	Every day	7 days	Android
P04^b^	Female	NH White	Some college, ND	Some days	30 days	Android
P05	Male	NH White	Some college, ND	Every day	7 days	Android
P06	Male	NH Black/AA^f^	Some college, ND	Every day	30 days	Android
P07	Male	NH Asian, NHPI^g^	HS equivalent	Every day	30 days	iOS
P08^b^	Female	Hispanic/Latino	Some college, ND	Every day	30 days	iOS
P09	Male	NH White	HS equivalent	Every day	30 days	iOS
P10^b^	Male	NH White	HS graduate	Every day	7 days	Android
P11^b^	Female	Hispanic/Latino	Some college, ND	Every day	30 days	iOS
P12^b^	Male	NH White	Some college, ND	Every day	7 days	Android
P13^b^	Female	NH White	HS graduate	Every day	30 days	Android
P14^b^	Female	NH White	2-year AD^h^	Some days	30 days	iOS
P15^b^	Female	NH Black/AA	HS incomplete	Some days	30 days	Android
P16^b^	Female	AIAN^i^	HS incomplete	Every day	30 days	iOS
P17^b^	Male	NH Black/AA	HS graduate	Every day	30 days	Android
P18	Female	NH Black/AA	Some college, ND	Every day	7 days	iOS
P19	Female	NH White	HS equivalent	Every day	7 days	Android
P20	Female	NH White	HS graduate	Every day	7 days	Android
P21	Female	NH White	Some college, ND	Every day	7 days	iOS
P22	Female	NH White	Some college, ND	Every day	30 days	iOS
P23	Male	NH White	Some college, ND	Every day	7 days	iOS
P24	Female	NH Black/AA	Some college, ND	Every day	30 days	Android
P25	Female	NH Black/AA	HS graduate	Some days	30 days	Android
P26	Male	NH Black/AA	Some college, ND	Every day	30 days	iOS
P27	Male	Hispanic/Latino	Some college, ND	Some days	30 days	Android
P28	Female	NH White	2-year AD	Every day	30 days	Android
P29	Female	NH White	Some college, ND	Every day	30 days	iOS
P30	Male	NH Asian, NHPI	HS graduate	Some days	6 months	Android
P31	Male	NH Black/AA	Some college, ND	Every day	6 months	Android
P32	Female	NH Asian, NHPI	HS graduate	Every day	30 days	iOS
P33	Male	Hispanic/Latino	2-year AD	Some days	6 months	iOS
P34	Male	NH Black/AA	2-year AD	Every day	6 months	Android
P35	Female	NH Black/AA	HS graduate	Every day	30 days	iOS
P36	Male	NH White	Some college, ND	Every day	7 days	Android
P37	Male	NH Black/AA	HS graduate	Every day	6 months	iOS
P38	Male	NH Mixed	HS graduate	Some days	7 days	iOS

a Includes unpublished data from Tesfaye et al, 2024; Wakeman et al, 2024.

b Participated in 2 focus groups.

c NH: Non-Hispanic.

d ND: no degree.

e HS: high school.

f AA: African American.

g NHPI: Native Hawaiian/Pacific Islander.

h AD: associate degree.

i AIAN: American Indian/Alaska Native.

We extracted 143 quotes across all semantic domains. A total of 110 quotes captured technology acceptance constructs and sentiment toward the smoking cues mobile game, whereas 33 quotes included suggestions to improve the game. Sentiment toward the smoking cues game was mixed, with negative (45/110, 40.90%) and neutral (36/110, 32.72%) quotes outweighing those with positive sentiment (29/110, 26.36%) (Table 3). Most quotes centered on performance expectancy (34/110, 30.90%), followed by hedonic motivation (17/110, 15.45%), facilitating conditions (10/110, 9.09%), and effort expectancy (6/110, 5.45%). Social influences on game acceptance and use were not discussed. Below we present illustrative quotes, with participant ID attribution, first on performance expectancy, then followed by all other technology acceptance themes as they were discussed to a lesser extent (Table S2 in Multimedia Appendix 1). Last, we present participants’ suggestions to improve the game’s functionality and design (Table S3 in Multimedia Appendix 1).

**Table 3.** Distribution of the number of quotes on smoking cues attentional bias modification mobile game by technology acceptance constructs and sentiment.

Themes	Sentiment			Total, n (%)
	Negative, n (%)	Neutral, n (%)	Positive, n (%)	
Effort expectancy	0 (0)	2 (33.33)	4 (66.67)	6 (5.45)
Facilitating conditions	4 (40.00)	5 (50.00)	1 (10.00)	10 (9.09)
Hedonic motivation	8 (47.05)	5 (29.41)	4 (23.52)	17 (15.45)
Performance expectancy	9 (26.47)	12 (35.29)	13 (38.23)	34 (30.90)
Social influence	0 (0)	0 (0)	0 (0)	0 (0)
Not applicable	24 (55.81)	12 (27.90)	7 (16.27)	43 (39.09)
Total	45 (40.90)	36 (32.72)	29 (26.36)	110 (100.00)

### Performance Expectancy

Perceptions of the smoking cues game’s usefulness were mixed in sentiment with 26.47% (9/34) of the quotes having a negative sentiment, 35.29% (12/34) neutral, and 38.23% (13/34) positive. Some participants attributed the usefulness of the game to its potential role as a distraction tool from cravings, which was rooted in participants’ familiarity with mobile games and their prior use of them as distraction tools.

*The distraction would be good and just having another thing to do and having my mind just getting distracted and not focusing on smoking. That’ll be helpful, really beneficial, really useful*.

[P06]

*I feel like it’d be very useful because I too kind of play games to distract myself, from negative thoughts, or habits*.

[P10]

Few participants who understood that the true purpose of the game was to modify unconscious attention to smoking cues emphasized the novelty of the concept and its potential usefulness in facilitating cessation.

*You’re kinda … psychologically … switching … I guess [the game] makes you less likely to focus on those things in real life. But I never really thought about that. I think that’s pretty cool*.

[P30]

*You’re using [the game] as a way to relocate your mind from a cigarette, basically, at that time that you need it the most*.

[P20]

Some participants expressed concerns over the smoking cues imagery, which could trigger cravings and be counterproductive to their quit attempts.

*I like the idea of a game, but I feel like it makes it kind of tough because … what if I’m playing this game and I see packs of cigarettes and stuff, and I’m like, oh, well, I could go for a cigarette now … Like, maybe trigger something that I wouldn’t already do … I like the idea of a game but maybe not necessarily having like smoking-related images*.

[P21]

*I almost feel like [the game is] trying to utilize … like cognitive behavioral therapy … where it’s like exposure therapy. But I think for people who are trying to not think about cigarettes, to think about them doesn’t necessarily help them*.

[P36]

Regardless of what participants thought the purpose of the game was, they wanted evidence of the effectiveness of the game in helping them quit smoking.

*I would have to see the game … in action I guess … to see … how enticing it is … and also know that it’s supposed to be proven to really help like psychologically and really impacts your brain functions as far as wanting to quit*.

[P27]

*The pictures [of the game] are … kinda lame. And I would want to like see … how that helps, because I think if that was just … a game, I would be like, what the heck is this. I wouldn’t understand without someone … explaining that it’s gonna actually help*.

[P11]

### Other Technology Acceptance Themes

#### Hedonic Motivation

Participants characterized the game as outdated, boring, and not fun, with 47.05% (8/17) of the quotes being negative in sentiment.

*I feel like the game seems just kinda boring*.

[P10]

The game fared worse in comparison to other mobile games that participants were accustomed to playing, especially as they highlighted enjoyability as an important user engagement factor.

*If I want to play a game and distract myself, I might just do that of my own accord. I just have a feeling that … the game within the app is probably not going to be as much fun as … whatever I was gonna play anyway, which is gonna distract me anyway*.

[P12]

*I think [the game] would have to be fun, but … not stressful either, because … I think a lot of times when some people play games, they want to smoke cause sometimes it can be a little bit stressful or boring, so it’d definitely have to be … very engaging and fun to actually want to play it and use that, and actually help*.

[P13]

#### Facilitating Conditions

Past experiences and attitudes toward mobile gaming, both positive and negative, affected participants’ perceptions of the game.

*[The game] reminds me of Fruit Ninja, but without flashing things, and I never really liked that game. That game frustrates me. So, this one probably would frustrate me too, which is the opposite of what I’m trying to do*.

[P28]

Additionally, compatibility of the game with their smart devices and the time demands to play the game were highlighted as possible barriers.

*If it’s not too … over the top, like, some people might not have … good enough phones to run [the game] … But as long as it doesn’t crash and is … decent then, yeah [I think the game would be easy to use]*.

[P13]

*I think it just depends on the audience, because if you’re not a gamer and you’re more into social media, and that’s you’re downtime … it just depends on how determined you are and if you’re game savvy and things like that*.

[P16]

#### Effort Expectancy

Six quotes referencing effort expectancy were generally positive in sentiment about the ease of playing the game (66.67%).

*I feel like [the game] would be a pretty easy to use feature*.

[P23]

### Suggestions

A total of 33 quotes included suggestions to improve the smoking cues game (Table S3 in Multimedia Appendix 1). First, participants suggested renaming the game to reference smoking.

*Definitely [the name of the game should reference smoking], if it’s going to be on a smoking app*.

[P09]

Because of its role in helping them quit, some participants suggested that the game playing time and scoring system should be linked to their quitting journey. For example, one participant suggested that players could score more points if they played the game when craving cigarettes.

*It might be cool, like, after a week of your set trigger times, like, you would only get points if you did … the little games at those trigger times … Instead of smoking a cigarette, you did a game. Instead of just someone playing games all day, getting points*.

[P21]

Because of concerns over cravings when seeing smoking-related images in the game, some suggested changing the premise of the game to enable players to “destroy” smoking stimuli instead of neutral stimuli.

*I think the game should be about … destroying cigarettes … instead of … choosing fruit over the cigarette, because … when you see cigarettes and you see the lighter … it’s … kind of giving me a craving, seeing the picture of cigarettes. So, I think it should be like something to destroy cigarettes, to show that it’s no good*.

[P25]

Drawing on their experiences with mobile games, participants suggested adding different levels to the game with varying difficulty (eg, increasing speed and time limits), competitive elements (eg, leaderboards), and the ability to invite friends to play the game as in multiplayer games.

*Maybe make it … like a high score kind of thing like old arcade games where you … have like a leaderboard and try to get the highest score and stuff. Just make it a … little competition, like friendly competition. Give it a little bit more of a distraction*.

[P05]

Other participants suggested adding other games to our cessation mobile app (eg, mindfulness games) to help individuals who smoke deal with smoking antecedents such as stress.

*Mindfulness might be better [for the game]. I think it would be maybe cool if … you picked like your stress, then you could have like a meditation. Or … if you’re like sad, you could have, like, a really cute game. Or, like something that would correlate to your mood to personalize it and … be like as helpful as possible*.

[P11]

## Discussion

### Principal Findings

This is one of the first studies, to our knowledge, that examines perceptions of a gamified ABM module within a multicomponent smoking cessation mobile app targeting individuals with low socioeconomic status who smoke [27,30]. As a novel concept, participants were willing to try the game provided there was evidence of its efficacy as a tool in aiding them to quit. However, only a few grasped the true purpose of the game as an attentional bias training tool, whereas most viewed the game as a distraction tool during cravings. Skepticism about the usefulness of the game was attributed to the possibility of triggering cravings upon the presentation of smoking cue imagery and the availability of other mobile games, more sophisticated in nature, that could distract individuals who smoke during cravings. Opinions were mixed concerning the game’s enjoyability but were largely positive for ease of use. Our findings underscore that the primary challenge to maximizing user acceptance is to communicate the purpose and benefit of an ABM game and to improve the game’s functionality and design elements to meet users’ expectations and withstand competition from commercially available entertainment games. This is particularly true if an ABM game were to engage users for an extended time that is needed to allow for the delivery of multiple training sessions to positively affect cessation. Beyond our ABM smoking cues game, these results could inform the development and user acceptability of gamified self-help ABM and CBM interventions for other substance use disorders and tobacco products. This is particularly relevant as CBM- and ABM-based mobile games and apps have generally had mixed effects on behavioral change [30]. Finally, our results highlight the importance of user-centered approaches for intervention development. User feedback will help us further improve our ABM mobile game by incorporating participants’ suggestions prior to evaluating its usability and efficacy in field studies.

### Comparison to Prior Work

Our approach to embedding an ABM mobile game within a multicomponent app for smoking cessation is novel. Past attempts to translate ABM techniques to mobile platforms as games have been limited to substance use disorders other than tobacco dependency (eg, opioid use) [49]. Few smartphone-based ABM interventions for smoking cessation have been standalone interventions. Furthermore, these interventions usually deliver the traditional visual probe test on a mobile platform rather than transforming the technique to embrace smartphones as a medium [27-29]. Our approach would allow for user-initiation of the training module at their own will and in their natural environments, eliminating one potential cause of the underperformance of traditional ABM interventions. This could be particularly beneficial to populations that cannot avoid or reduce exposure to smoking-related imagery per clinical guidelines for treating tobacco dependency [11]. Given the scarcity of evidence on ABM mobile games for smoking cessation in general and with disadvantaged groups in particular, research is needed to examine their efficacy in reducing smoking and its antecedents (eg, cravings and reactivity to smoking cues).

Additional strengths of our game-based approach to smoking cues ABM include the accessibility and familiarity of smartphones and mobile games, particularly among young and middle-aged adults [23,50]. Indeed, most participants in our study agreed the game was easy to use and referenced prior experiences with mobile games. The downside to this approach is that it excludes by default individuals with certain predispositions, thus limiting the efficacy of smoking cessation intervention with an ABM component. For example, less digitally literate and older individuals might not be willing or able to use mobile health games [50]. More importantly, high user expectations of ABM game functionality and design present a fundamental challenge. Mobile health games must compete with commercially available, professionally developed entertainment games for users’ attention and engagement, especially as most mobile apps rapidly lose users after their first use [51]. While smoking cessation interventions are on average 6 weeks long, sustaining engagement during this period is a challenge. However, limited evidence suggests that the use of a game was positively associated with the number of smoking cessation app sessions and time spent on the app [52,53].

Indeed, enjoyability emerged as a main factor in user acceptance of the ABM game. Past work has established the important role of enjoyability in users’ adoption of new technologies as well as its impact on user engagement for serious games [35,54]. User engagement, in turn, has been associated with abstinence in pilot testing [55]. Accordingly, negative sentiments in discussing the game’s level of fun and enjoyment are concerning. Some remarked that the game appeared outdated, which may be on account of seeing an early prototype or on account of having experiences with increasingly sophisticated mobile games. Partnerships between academics and mobile game developers may be a beneficial approach to elevate health games [52]. Our game’s level of enjoyability could be improved by implementing user suggestions, which primarily focused on adding new elements or approaches commonplace in other games. Several of the suggested additions (eg, leaderboards, levels, and rewards) have been desired by individuals who smoke in other work and successfully implemented in both smoking cessation and ABM apps [30,56-58].

Our findings show that communicating the intent and benefit of an ABM is a primary requisite to user acceptance. Skepticism of the game’s performance stemmed primarily from concerns that the presentation of cue imagery could trigger cravings and from perceptions of the game as a distraction tool, in which case preferable alternatives are readily available. Suggestions to redesign the game to allow users to “destroy” smoking-related images as well as the common use of mobile apps and games for distractions echo our participants’ perceptions of the ABM game [57,59]. Previous research on an ABM app for substance use disorder documented similar results [56]. Moreover, cravings in response to cues are well-documented among individuals who smoke and provide credence to participants’ concerns [60]. However, the game requires focusing one’s attention on neutral images (ie, fruit slices) to score. For the ABM to be effective, it is necessary to use stimuli that are relevant to and associated with users’ attentional biases [61]. Past qualitative research has identified the need for users to understand the rationale behind CBM and ABM [62,63], which is critical for engagement [54]. Thus, it is imperative to identify appropriate means to communicate the rationale behind the game to users and its potential benefit in helping them quit. This could potentially be accomplished by providing a written rationale, written instructions, or informational tooltips, as some participants suggested. Future research is needed to field test the effects of an ABM game on intended (eg, modifying attentional biases) and unintended (eg, triggering cravings) outcomes.

### Strengths and Limitations

This study has several strengths, including the recruitment of a diverse sample of participants with low socioeconomic status with roughly ≤50% of the participants from one race or sex. Furthermore, our qualitative methodology provides a wealth of information on user perceptions of the ABM mobile game under investigation. Nonetheless, this study has several limitations. First, although the moderator assured participants that there were no right or wrong answers, our results could have been influenced by social desirability bias [64]. Second, our focus groups were held during the COVID-19 pandemic, which resulted in participants seeing web-based mock pages of the game rather than using it had the discussions been conducted in person. Participants were shown an early prototype of the smoking cues mobile game, which may have affected their perceptions. Our results may have been impacted by the high exposure to and familiarity with mobile games that young adults have, which may not be representative of all individuals who smoke [50]. However, we will field test our smoking cessation app to determine whether the ABM game significantly contributes to quit rates among adults older than 18 years and to shed light on their experiences using the game in their natural environment, which will complement this formative work. Last, although the intercoder agreement for technology acceptance and sentiment domains was low, all inconsistencies were resolved through discussions between MW, LT, and SEL.

### Conclusions

This study is one of the first to characterize users’ perceptions of an ABM mobile game for smoking cessation. Participants had mixed perceptions of the game’s usefulness and enjoyability but were willing to use it provided evidence of its efficacy in aiding smoking cessation. These results will help guide the development and improvement of the game to support adults with low socioeconomic status who smoke. Further work should explore the real-life use and effects of gamified ABM techniques in general and for smoking cessation in particular both independently and as part of multicomponent behavioral interventions.

## Supplementary material

10.2196/59515 Multimedia Appendix 1 Supplementary files.

## Abbreviations

ABM: attentional bias modification
CBM: cognitive bias modification
