# Supplementary material for: Perceptions Toward an Attentional Bias Modification Mobile Game Among Individuals With Low Socioeconomic Status Who Smoke: Qualitative Study
Source: JMIR Serious Games. 2025 May 9;13:e59515. doi: 10.2196/59515 (PMC12083738; doi:10.2196/59515)
Supplement: Multimedia Appendix 1 [file games-v13-e59515-s001.docx]

**Note S1. Screening survey**

DATE [MM/DD/YY] ………………….

Please answer the following questions. If you appear to qualify, we will follow up with you by email or telephone to schedule the test session. Your answers to these pre-qualification questions and all information you provide during the subsequent screening call and eventual test session will be treated as strictly confidential. Your name, contact information, and other personally identifiable information will not be shared with our client or anyone else. Data will be reported only in aggregate or after information that would identify you personally has been removed and replaced with a unique ID. We will not store your name or contact information in the same files with the other information you provide.

*We would like to learn about your background*

1. Which of the following best describes your age?

1 17 and younger

2 18 to 29 years old

3 30 to 39 years old

4 40 to 49 years old

5 50 to 59 years old

6 60 and older

2. With what gender do you identify?

1 Male

2 Female

3 Other (please specify ---------)

8 Refused to answer

3. Do you consider yourself Hispanic or Latino?

0 No

1 Yes

4. Which of the following includes your racial identity? (One or more categories may be selected)

1 White

2 Black or African American

3 American Indian or Alaska Native,

4 Asian Indian, Chinese, Filipino, Japanese, Korean, Vietnamese, other Asian

5 Native Hawaiian, Guamanian or Chamorro, Samoan, other Pacific Islander

6 Other (please specify ---------)

5. What is the highest grade or level of schooling you completed?

0 Less than high school (Grades 1-8 or no formal schooling)

1 High school incomplete (Grades 9-11 or Grade 12 with NO diploma)

2 High school graduate (Grade 12 with diploma or GED certificate)

3 Some college, no degree (includes some community college

4 Two-year associate degree from a college or university (e.g., AA, AS)

5 Four-year college or university degree/ Bachelor’s degree (e.g., BS, BA, AB) [Terminate]

6 Some postgraduate or professional schooling, no postgraduate degree (e.g., some graduate school) [Terminate]

7 Postgraduate or professional degree, including master’s (for example: MA, MS, MEng, MEd, MSW, MBA), doctorate (for example: PhD, EdD), medical or law degree (for example: MD, DDS, DVM, LLB, JD) [Terminate]

6. Are you currently enrolled in school?

0 No, not enrolled (skip next question)

1 Yes, enrolled

8 Refused to answer

9 Do not know

7. What school?

1 High school

2 Technical, trade, or vocational school

3 2-year college (undergraduate)

4 4-year college (undergraduate) [Terminate]

5 Graduate school [Terminate]

*Next, we have a few questions about your smoking behavior. We know that this information is personal, but please remember your answers will be kept confidential*

8. Have you smoked at least 100 cigarettes (5 packs or more) in your entire life?

0 No [Terminate]

1 Yes

9 Do not know [Terminate]

9. Do you now smoke cigarettes every day, some days, or not at all?

1 Every day

2 Some days

3 Not at all [Terminate]

9 Do not know [Terminate]

10. Do you plan to ever quit smoking for good?

0 No (skip next question) [Terminate]

1 Yes

9 Do not know [Terminate]

11. When do you plan to quit for good?

1 In the next 7 days

2 In the next 30 days

3 In the next 6 months

4 In the next year [Terminate]

5 More than one year from now [Terminate]

9 Do not know [Terminate]

12. Which of the following tobacco products do you currently use? Please check all that apply.

1 Cigarettes

2 Cigars [Terminate]

3 Little cigars or cigarillos [Terminate]

4 Tobacco is a hookah [Terminate]

5 E-cigarette or other vaping device

6 Chewing tobacco, snus, snuff, or dip [Terminate]

7 Pipe filled with tobacco [Terminate]

13. Are you currently … (Please check all that apply)

1 Using any smoking cessation medications [Terminate]

2 Receiving any smoking cessation counseling support [Terminate]

3 Using any smoking cessation complementary or alternative methods [Terminate]

4 Using any tobacco or nicotine delivery systems [Terminate]

5 Trying to quit on your own

6 Enrolled in a smoking cessation study [Terminate]

14. Please indicate what type of cell phone you have.

1 iPhone

2 Android

3 Blackberry

4 Windows phone

5 Basic cellphone only [Terminate]

8 Refused [Terminate]

9 Do not know [Terminate]

Participants who are not eligible will receive the following message:

Thank you for participating in our questionnaire but you are not eligible for this study. There are many possible reasons why people are not eligible. These reasons were decided earlier by our client. However, thank you for your interest in this study and for taking the time to answer our questions.

For free help on quitting smoking:

Call: 1-800-QUIT-NOW

Visit: [www.smokefree.gov](http://www.smokefree.gov)

Eligible participants will receive the following message:

Thank you for participating in our questionnaire! You are eligible to continue to the next part of the study. Please provide your email address and cellphone number in the space below. We will contact you with information on next steps! Thank you again for your time!

Name: ……………………..

Your email address: ……………………..

Your cell phone number: ……………………..

Preferred method of contact: ……………………..

1 Email

2 Phone call

3 Text messages

**Note S2. Moderation guide.**

Introduction

The sponsors of this focus group developed Quit Journey, a smoking cessation mobile application, to help those who want to quit smoking. Imagine that we are the committee charged with the task of giving the sponsors feedback on the app. I’d like to describe features of this app and get your reactions.

The app includes a video game called “Fruit Squish.” The game is intended to help smokers focus less on things that remind them of smoking. The idea is that smokers tend to focus their attention on smoking items such as ashtrays and this happens without them being aware of it. In the game, we will show users smoking items such as a lighter or cigarette pack and ask them to click on a fruit that appears on the opposite side of the phone screen. Similar to spot-the-dot games. Over time, this training will help them focus less and less on smoking-related items they come across in their natural environment.

Questions

1. What is your general reaction to this idea? Is there anything you especially like about it? Is there anything you especially dislike? Is anything confusing?
2. What do you think of these app’s screenshots? What do you suggest to improve them?
3. What do you like about it? What do you dislike about it? What do you suggest to improve it? How useful would the app be to you with this feature? Do you think the app with this feature would be fun to use? Do you think the app with this feature would be easy to use?

**Table S1. Consolidated criteria for reporting qualitative studies (COREQ).**[45]

| Topic | Guide Questions/Description | Page No. |
| --- | --- | --- |
|  |  |  |
| Domain 1: Research team and reflexivity |  |  |
|  |  |  |
| *Personal Characteristics* |  |  |
| Interviewer/facilitator | Which author/s conducted the interview or focus group? | 6 |
| Credentials | What were the researcher’s credentials? | 1 |
| Occupation | What was their occupation at the time of the study? | 6 |
| Gender | Was the researcher male or female? | 6 |
| Experience and training | What experience or training did the researcher have? | 6 |
|  |  |  |
| *Relationship with participants* |  |  |
| Relationships established | Was a relationship established prior to study commencement? | 6 |
| Participant knowledge of the interviewer | What did the participants know about the researcher? | 6 |
| Interviewer characteristics | What characteristics were reported about the interviewer/facilitator? | 6 |
|  |  |  |
| Domain 2: Study design |  |  |
|  |  |  |
| *Theoretical framework* |  |  |
| Methodological orientation and Theory | What methodological orientation was stated to underpin the study? | 6 |
|  |  |  |
| *Participant selection* |  |  |
| Sampling | How were participants selected? | 6 |
| Method of approach | How were participants approached? | 6 |
| Sample size | How many participants were in the study? | 6 |
| Non-participation | How many people refused to participate or dropped out? | 6 |
|  |  |  |
| *Setting* |  |  |
| Setting of data collection | Where was the data collected? | 6 |
| Presence of non-participants | Was anyone else present besides the participants and researchers? | 6 |
| Description of sample | What are the important characteristics of the sample? | 8-9 |
|  |  |  |
| *Data collection* |  |  |
| Interview guide | Were questions, prompts, guides provided by the authors? Was it pilot tested? | 6 |
| Repeat interviews | Were repeat interviews carried out? If yes, how many? | 6 |
| Audio/visual recording | Did the research use audio or visual recording to collect the data? | 6 |
| Field notes | Were field notes made during and/or after the interview or focus group? | 6 |
| Duration | What was the duration of the interviews or focus group? | 6 |
| Data saturation | Was data saturation discussed? | 6 |
| Transcripts returned | Were transcripts returned to participants for comment and/or correction? | 6 |
|  |  |  |
| Domain 3: Analysis and findings |  |  |
|  |  |  |
| *Data analysis* |  |  |
| Number of data coders | How many data coders coded the data? | 7 |
| Description of the coding tree | Did authors provide a description of the coding tree? | 7-8 |
| Derivation of themes | Were themes identified in advance or derived from the data? | 6-7 |
| Software | What software, if applicable, was used to manage the data? | 7 |
| Participant checking | Did participants provide feedback on the findings? | 7 |
|  |  |  |
| *Reporting* |  |  |
| Quotations presented | Were participant quotations presented to illustrate the themes/findings? Was each quotation identified? | 11-14 |
| Data and findings consistent | Was there consistency between the data presented and the findings? | 11-14 |
| Clarity of major themes | Were major themes clearly presented in the findings? | 11-14 |
| Clarity of minor themes | Is there a description of diverse cases or discussion of minor themes? | 11-14 |

**Table S2.** **Themes and illustrative quotes of individuals who smoke related to an attentional bias modification smoking cues game by technology acceptance factors and sentiment.**

| **Technology acceptance theme** | **Quotations** | **Sentiment** |
| --- | --- | --- |
| Performance Expectancy | P10: [The game seems] not necessarily that useful. | Negative |
|  | P34: This game, in particular, probably wouldn't be a good one, maybe … So, they show you images … and you have to just click on it and … something like that. If you're just trying to … change people's habits, I guess. But if you're just trying to just play a game, just to play a game, that's what this thing feels like. Just a regular game. Not really tailored to the app. | Negative |
|  | P11: The pictures [of the game] are … kinda lame. And I would want to like see … how that helps, because I think if that was just … a game, I would be like, what the heck is this. I wouldn't understand without someone … explaining that it's gonna actually help.^a^ | Negative |
|  | P37: [The game] feels … to me kind of more of a distraction than a help. | Negative |
|  | P31: [The game] wouldn't distract me at all, they [are] just pieces of fruit … it didn't work for me at all. | Negative |
|  | P36: I almost feel like [the game is] trying to utilize … like cognitive behavioral therapy … where it's like exposure therapy. But I think for people who are trying to not think about cigarettes, to think about them doesn't necessarily help them. | Negative |
|  | P21: I like the idea of a game, but I feel like it makes it kind of tough because … what if I'm playing this game and I see packs of cigarettes and stuff, and I'm like, oh, well, I could go for a cigarette now … Like, maybe trigger something that I wouldn't already do … I like the idea of a game but maybe not necessarily having like smoking-related images. | Negative |
|  | P22: I don't know the science behind … seeing a cigarette [and] an ashtray and playing a game where you're seeing all of these things is supposed to help you … If it’s building my tolerance or something, but if that was like meant to distract me, I think that would be … kind of a toxic thing for distracting you, just seeing all the things that remind you of it. | Negative |
|  | P24: If it's just the setting of a cigarette and ashtray, like little pictures and stuff … I thought it was … more like … a weird, interactive game like, oh, see how long you were … distracted … So, if it’s just photos or just like a reminder then no, I don't think the game’s that necessary anymore … There's too many options. Like, I'm supposed to stay focused. | Negative |
|  | P27: I would have to see the game … in action I guess … to see … how enticing it is … and also know that it's supposed to be proven to really help like psychologically and really impacts your brain functions as far as wanting to quit. | Neutral |
|  | P28: The game has to be engaging enough for me to not go back to thinking how I was thinking previously. But I like that it's a game and I'd be willing to try it … just to see what it's like, if it actually worked.^b^ | Neutral |
|  | P03: I would say [whether the game is useful] really just depends on the person … Personally, I don't like to play video games, but my mom, on the other hand, she's more of the type of person that will sit there and want to play … for a couple hours or something … so it might be more helpful for her … versus me or my stepdad, because we're more active people … It really would just depend on the person and how active they are in their life, and what they have going on. | Neutral |
|  | P01: I'm not sure that [the game] would be helpful for me, but maybe for other people. | Neutral |
|  | P14: I don't know that it would be extremely helpful for my personal preferences in what I feel I would be likely to use and feel is … really helping me. | Neutral |
|  | P20: I'm not really sure about that game in particular … What if … I wasn’t having a trigger or really thinking about a cigarette … and then I started playing the game and it did … something. | Neutral |
|  | P04: Everybody plays games, so I think it's a great idea for having a game if you want to smoke and you're like, oh, I want to just play this game instead. But, if I'm … trying to get away from a craving and then all of a sudden, I see like a cigarette on the screen … and I’m supposed to go pick a fruit, then I might just be like, oh, that cigarette looks nice, too. | Neutral |
|  | P13: I mean, I think [the game is] a cool idea if it actually works … It seems like, oh, like, you have to go the opposite way, but it's still showing … the cigarettes and stuff … I play a lot of games, and you do focus on enemies a lot in the game and that would kind of be the same thing, like focusing on that to stay away from it … I don't know how that would … relate to real life, but it's a cool concept, I guess. Might make it more fun. | Neutral |
|  | P05: Yeah, [the game is] good in theory for sure … Some people, especially a younger crowd, they’re really into the … smart phone games and stuff like that … It might work for some, may not for others, but having so many different options and different ways to give incentive and stuff … in this app, I like, because it seems like overall, there's something for everyone in there … Some people the game … may not help too much. Some people … might find that helps them a lot. So, just having another option, though, I don't see any harm. | Neutral |
|  | P11: I would try [the game]. I don't know how useful it would be, because … I've never done it before. But … it would be cool to have an app that … would be accessible to try, if … does have data that shows that this … distraction technique works.^b^ | Neutral |
|  | P22: If it's been shown that [the game is] like helpful, I don't know. | Neutral |
|  | P11: I think [the game] could be helpful, but I don't know … if it was, that would be awesome. | Neutral |
|  | P30: You're kinda … psychologically … switching … I guess [the game] makes you less likely to focus on those things in real life. But I never really thought about that. I think that's pretty cool.^c^ | Positive |
|  | P17: I like [the idea of a smoking cues game]. I think it’d help us focus and get us try not to smoke. | Positive |
|  | P16: [A smoking cues game is] a good idea. I had never played spot the dot, so I'm not really sure what that will look like …. When Candy Crush came out, a lot of people would focus on that. I knew I was really focused on that, and … I notice a lot of times when I have something to focus on, I do smoke less cigarettes and when I'm super bored, have nothing to do, I smoke more. So, I think … the idea of that is good. | Positive |
|  | P21: I think it would be most useful to play [the game] during cravings, but, at the same time, I guess doing [it during] an idle time would be a good idea … because … you don't know … you're gonna get a craving. | Positive |
|  | P20: I think [having a game is] still a good idea. Like … having … little mini games … very passive … Candy Crush or like … the little ball shoot games … matching games, Bejeweled, you know just stuff like that to distract you … I think those are still really important key factors in the game concept. | Positive |
|  | P20: You're using [the game] as a way to relocate your mind from a cigarette, basically, at that time that you need it the most. | Positive |
|  | P06: The distraction would be good and just having another thing to do and having my mind just getting distracted and not focusing on smoking. That'll be helpful, really beneficial, really useful. | Positive |
|  | P24: A game would be kinda distracting … it would help, because now, [if] I notice if the packs of cigarettes were in the house, they would probably just all be gone … So, the more I’m playing a game, I feel like I wouldn't want to … be idle right after that, like go smoke another cigarette … I would be like, okay, that was enough idleness, let me go back to doing something productive. | Positive |
|  | P10: I feel like it'd be very useful because I too kind of play games to distract myself, from negative thoughts, or habits. | Positive |
|  | P09: [A smoking cues game] would be cool because it keeps your mind preoccupied. I know games are helpful for me when I am trying to keep my mind off of things. | Positive |
|  | P08: I think [playing a smoking cues game would be] really cool, actually because it would … take your mind off of what you would typically be doing at that time. | Positive |
|  | P17: [The game would] kinda distract you from smoking too. | Positive |
|  | P18: You get to play the game and it helps like keep your mind off smoking or remind you of the effects of smoking or whatever the case may be. | Positive |
| Hedonic Motivation | P28: I really dislike the game. I feel like it’s outdated and not fun, it's more stressful than anything. | Negative |
|  | P35: [The game is] extremely boring … I would not play that at all … it's not interactive at all. It just seems like your two-year-old and you're trying to like relate words, like images to words or something like that. No … it doesn't catch my attention.^b^ | Negative |
|  | P11: I agree, [the game is] pretty boring. | Negative |
|  | P10: I feel like the game seems just kinda boring. | Negative |
|  | P37: This game, to me, kind of feels like it's just filling a space, rather than … trying to engage you with a fun game. It's just kind of like, we have a game. | Negative |
|  | P01: I’ve gotta be terribly bored [to play the game]. | Negative |
|  | P03: I've got to be like excruciatingly bored and I have nothing else to do, nothing on my TV, apps, or anything like that … nothing else to do for me [to] want to … play a game, period … And even then, to be truthfully honest with you, 9 times out of 10 it won't even be me probably playing the game, it'll probably be my nephew.^b^ | Negative |
|  | P12: If I want to play a game and distract myself, I might just do that of my own accord. I just have a feeling that … the game within the app is probably not going to be as much fun as … whatever I was gonna play anyway, which is gonna distract me anyway. | Negative |
|  | P24: I just feel like the game is, I don't know, it's personalized, but then the same time, it doesn't sound exciting. | Neutral |
|  | P13: I think [the game] would have to be fun, but … not stressful either, because … I think a lot of times when some people play games, they want to smoke cause sometimes it can be a little bit stressful or boring, so it’d definitely have to be … very engaging and fun to actually want to play it and use that, and actually help. | Neutral |
|  | P37: Yeah, the game, it could be fun. | Neutral |
|  | P13: It depends on if the game’s fun or not. Like, if it's fun, then yeah, I’d definitely find it very useful, but if it's a boring or something, I'd probably just want other options. | Neutral |
|  | P22: I think maybe [the game would be fun] for some people, but I don't think it would be useful or that fun for me. | Neutral |
|  | P17: I like [the game]. It’s like … the fruit game, but … it has something to do with smoking. I think it'd be fun and a good thing to pass time when you're thinking about smoking. | Positive |
|  | P18: This [game] will make it fun … like it’ll just be fun. | Positive |
|  | P11: I think having the option’s good, [the game] will be fun for some people. I think I would definitely … check it out, and if I did enjoy it, then I'd be happy it was there.^b^ | Positive |
|  | P17: [The game] sounds fun, worth a try.^b^ | Positive |
| Facilitating Conditions | P28: [The game] reminds me of Fruit Ninja, but without flashing things, and I never really liked that game. That game frustrates me. So, this one probably would frustrate me too, which is the opposite of what I'm trying to do. | Negative |
|  | P17: I don’t like playing games anyway. | Negative |
|  | P23: I don't think [the game] would be useful for me because … I also don't play very many games on my phone. So, I don't think I would use it very much.^b^ | Negative |
|  | P22: I probably wouldn't use it, to be honest … I don't play that many games on my phone anyway, but the ones I do play, those are … the ones I like and that I play.^b^ | Negative |
|  | P28: This is something I already tried to do with like Candy Crush, you know, to get my mind off of things [using a game]. | Neutral |
|  | P03: I don't know, personally, because … personally, I'm not really a game person, you know, like I've done the Candy Crush and all of that … and it just got old, real fast, for me. | Neutral |
|  | P14: I don't know … I'm not a big game person. I mean I have played like Candy Crush and things like that. | Neutral |
|  | P16: We also have to factor … some people work … 9 to 5 so …. after they get off work and get the kids ready or get in bed … I don't think they're going to want to focus on … their phone. I mean a lot of us do. We do go … straight to social media as we lay in bed, and I know I do, and I know a lot of people that do. So, I think it just depends on the audience, because if you're not a gamer and you're more into social media, and that's your downtime … it just depends on how determined you are and if you’re game savvy and things like that. | Neutral |
|  | P13: If it's not too … over the top, like, some people might not have … good enough phones to run [the game] … But as long as it doesn't crash and is … decent then, yeah [I think the game would be easy to use]. | Neutral |
|  | P30: I've never really thought about [a smoking cues game] but that sounds really, really interesting. | Positive |
| Effort Expectancy | P21: I think [the game being easy to use] depends on the layout of it … how it's kinda laid out on the app itself.^a^ | Neutral |
|  | P11: As long as … [the game] functions and doesn't … crash, if the app all works well then it would be easy to use. | Neutral |
|  | P20: [The game] should be [easy to use], yes, most definitely. | Positive |
|  | P05: [The game] sounds pretty simple. | Positive |
|  | P22: [The game] sounds easy. | Positive |
|  | P23: I feel like [the game] would be a pretty easy to use feature. | Positive |
| Not Applicable | P29: I like the game the least [of the new features]. I like the concept of having a game, but I think the game does need a bit of work. | Negative |
|  | P13: I personally just didn’t like the game. I don't think I would really want to play this too much, because it's kind of just pressing things. I get the idea of it. I just wouldn’t play it, I don't think.^b^ | Negative |
|  | P01: [The game] doesn’t seem very interesting. | Negative |
|  | P29: I know it's just a prototype, but [the game] does look … a little outdated, so that wouldn't make me want to play it.^a^ | Negative |
|  | P28: I feel like the game is a little bit slow and … outdated.^a^ | Negative |
|  | P13: The thing I like least about the app is the game. | Negative |
|  | P30: I don’t really know what I like the least [about the app]. I'd probably have to say the game as well. | Negative |
|  | P26: I think … the only change for me was just the game. I think everything else is [in the app] great. | Negative |
|  | P26: I didn't like the game … I couldn't wrap my head around that. | Negative |
|  | P08: I haven't played [the game] so I don't understand the concept of it. But I don't know if that's something I would ever want to play, actively. And I also don’t really like the name because it doesn't have anything to do with smoking. So, if I had a first look at the name, I would have no idea that it's toward smoking.^b^ | Negative |
|  | P12: I don't like the name of it … Fruit Squish. | Negative |
|  | P14: I don't really like the name [Fruit Squish] either. | Negative |
|  | P12: To me, it looks … like you gotta click the fruit, like, it's gonna pop up on the screen and see how fast you can click the one and not click the other. But then when I look at this … the cigarettes … at first it looked like a box of French fries to me. So, that's kind of confusing. But that’s just maybe ‘cause [the screenshots are] too little right now. | Negative |
|  | P12: [The thing about the app I like the] least would be the game. | Negative |
|  | P32: I think the game’s fine, but the name [Fruit Squish] is pretty weird. | Negative |
|  | P13: I don’t really like [the name Fruit Squish]. | Negative |
|  | P12: [The name Fruit Squish is] very generic and … it just sounds kind of boring the first time I hear it. | Negative |
|  | P12: I don't like [the name Fruit Squish]. | Negative |
|  | P13: I think [the name Fruit Squish is] kind of generic … I also think it's kinda like a childish, and kinda sounds a little bit disgusting to me as well. | Negative |
|  | P13: I don't even care that's [the game is] kinda childish … I think [the name Fruit Squish] sounds kind of disgusting … I just think I don’t really like the whole squish thing. | Negative |
|  | P12: From the [game’s] name you don't see how it relates to the quitting … it seems … kind of unrelated. | Negative |
|  | P22: I don't know. [The game] feels really weird and like for a little kid, except a little kid isn't smoking. | Negative |
|  | P23: A kid could just like stumble across that [game and] … maybe fall into smoking that way … A game … wouldn't be best. | Negative |
|  | P22: I feel like that [game] seems like torture. | Negative |
|  | P29: I think … [the game is] a cool concept, but it doesn't seem super engaging. | Neutral |
|  | P34: It just really depends on the game and how it's put together. I feel like … it can be a good idea, but … it just depends on the game and the messages inside the game. | Neutral |
|  | P02: I'm not always on videogames, it's not really engaging as it used to be. | Neutral |
|  | P16: Maybe [the game] should be an option … I think it should be an option for those that are game savvy because you never know like you might like the game, you might not. | Neutral |
|  | P04: I mean, I don't hate the name [Fruit Squish]. | Neutral |
|  | P12: I don't know if I would want to play [the game] just … for its own sake. | Neutral |
|  | P11: I think [the name Fruit Squish is] fine. It sounds kinda like Fruit Ninja. | Neutral |
|  | P11: [The name Fruit Squish is] childish, but I think … if it's gonna be like a game … then it's like fine to have. Then, it shows it’s a game instead of like another serious thing like all the other things are on there … Maybe having it be … kinda childish might be kind of good, like, oh, it's something you can do, that's not so serious. | Neutral |
|  | P23: I don't know how I feel about that. Yeah, it kind of seems like a lot, a game …Yeah, I just think a game would be a little too much. | Neutral |
|  | P22: I think [Fruit Squish is] like a silly name … It makes it sound like fun and cutesy but then there's kinda … this dark stuff in it with these cigarettes that you're seeing and ashtrays … It does sound like this little kid, cutesy game by the name. | Neutral |
|  | P15: I play games like every now and then, but I don't know. I don't stay focused on the game for too long though. I'm not really sure. It might be a good idea, though. | Neutral |
|  | P09: [The game’s name is] not related to smoking cigarettes by any means. Sounds like the other game that's really popular. | Neutral |
|  | P14: I like the idea [of a game] as well. I think it's great. | Positive |
|  | P21: Yeah, I think [the game] would be a really good idea. | Positive |
|  | P20: I think [the name Fruit Squish is] cute and it's catchy. | Positive |
|  | P21: I think [Fruit Squish is] a cute name too. | Positive |
|  | P05: I like that for a quitting app, [the name Fruit Squish is] just kind of lighthearted and not … super intense or serious, it's not like it makes you feel … bad about using an app to quit smoking or bad about your habit. It's kinda cute and fun. | Positive |
|  | P12: Yeah, [a smoking cues game is] a fine idea. | Positive |
|  | P09: I’d play the game too, on my breaks or something like that.^b^ | Positive |

Participant ID appears before each quote for attribution.
^a^Indicates quote references design concepts.
^b^Indicates quote references intent/willingness to use.
^c^Indicates quote references novelty.

**Table S3.** **Illustrative quotes of individuals who smoke related to suggestions for improving an attentional bias modification smoking cues game.**

| **Category** | **Quotations** |
| --- | --- |
| Game Elements | P09: If the game gives me rewards as well that would definitely keep me coming back to that app and make me more use out of the app as well. Keep me more engaged with it. |
|  | P09: So maybe a structure [for the game] around Super Mario Bros … but keeping in mind that you're there to keep your mind off smoking and you're gonna get rewards for being there, not for smoking a cigarette and playing the game, rather. |
|  | P07: I think it's called Fruit Ninja, you have to like slice the fruits or whatever, but what if … instead of fruit flying up, … it's like cigarettes, and … it's kind of like the same concept, but cig smash or something … a bunch of cigarettes flying up in the sky and you have to … blow them up or cut them up or … something cool. |
|  | P04: I think [it would] be good to have a different level than that. Higher the level … the more intense it gets because I love brain games. So, the more I have to think or … the quicker I have to think about something, or you know react to it, I enjoy. So, I would enjoy … the levels getting harder as it goes. |
|  | P05: Maybe make it … like a high score kind of thing like old arcade games where you … have like a leaderboard and try to get the highest score and stuff. Just make it a … little competition, like friendly competition. Give it a little bit more of a distraction. |
|  | P06: Maybe … set some goals for every day and the player should try to achieve those goals every day. That will motivate the player to play [the game] daily. |
|  | P21: Would say definitely [have] different levels [for the game] so you feel like you're achieving something, so you feel like … it's not just … harder in difficulties … You feel like you're getting better at the game and that it's worth playing as opposed to just being too easy or too hard … You want that … happy medium. |
|  | P20: I agree [there should be multiple levels in the game] … I feel like … there should be some that are relaxed and … passive and then some that are … not just levels, but … they're more kind of like Flappy Bird … How like, you can't wait to get to the next one. |
|  | P25: I think the game should be about … destroying cigarettes … instead of … choosing fruit over the cigarette, because … when you see cigarettes and you see the lighter … it's … kind of giving me a craving, seeing the picture of cigarettes. So, I think it should be like something to destroy cigarettes, to show that it's no good. |
|  | P26: I think maybe because of the display and when I get an in-action gameplay … but maybe I think a different design for the game …Yeah, that’ll probably the only thing, actually. I can't think of any ideas to give as far as a game that could be … much fun to take away the thought of smoking. |
|  | P04: I think it would be cool if [the game] had maybe different levels. Like you start out one level slower or you just tap on it and then maybe it starts falling or it just starts getting quicker the more you go … Give your brain a real tease, you know? |
|  | P02: I don't know how engaging [the game] is. I think it should be a little faster. Something to really, really distract. |
|  | P29: Maybe adding a timer … would make [the game] more fun. |
|  | P21: Yeah, and have [the game] be [worth] … more points at … your trigger times. |
|  | P30: The game itself … can be improved a little bit. Maybe, if you're given like a time limit or something to press as fast as you can. |
|  | P13: Maybe you could have like puzzles or something as a game. People really enjoy doing … puzzles and you can put the concept of the not smoking into that somehow. Maybe that might be more engaging to a lot of people, sort of like … Lily's Garden or something like that kind of puzzles. |
|  | P13: The big thing that I would really want change is probably the game. To me, it doesn't even look like it fits the app too much, and the game is kind of like outdated … and it doesn't seem fun at all. And maybe adding like, the competition or being able to add friends … mainly just the game, and maybe adding friends would be nice. |
|  | P11: Mindfulness might be better [for the game]. I think it would be maybe cool if … you picked like your stress, then you could have like a meditation. Or … if you're like sad, you could have, like, a really cute game. Or, like something that would correlate to your mood to personalize it and … be like as helpful as possible. |
|  | P36: I think it'd be cool … if somehow you could … either send people to the games on their own phone or there's like some gaming platform I used once that was … to … increase your attention or … just like your focus. Like, even just … mindfulness games might be more helpful. |
| Timing | P09: [For the frequency of using the game,] just whenever you feel that craving, then instead of picking up that cigarette, you can run to the app. |
|  | P17: In the evening [would be the best time to play the game], you don’t really have nothing to do. |
|  | P10: If there's a pop up for the game when I have a craving, I feel it could be very useful for me to keep off cigarettes. |
|  | P10: I think whenever you have a craving it'd be good to play [the game] to kinda distract yourself. |
|  | P05: I would say [to play the game] at least once if the purpose of it is to kind of train your mind at least once a day even if you just pick it up for five minutes or so. It would really depend on the person and their schedule. |
|  | P13: I think it would be cool if [the game] like came up … during your craving times or times that you're most likely smoke. Just to have something small to do so you don't think about that. That might be useful. |
|  | P21: I don't know when [a craving is] going to happen. So … [how many times to play the game] would just have to be personalized and what each person feels is best for themselves |
|  | P21: It might be cool, like, after a week of your set trigger times, like, you would only get points if you did … the little games at those trigger times … Instead of smoking a cigarette, you did a game. Instead of just someone playing games all day, getting points. |
| Other | P09: Definitely [the name of the game should reference smoking], if it's going to be on a smoking app. |
|  | P11: If [the game] had … some like small text under the name that was like, “a game to help distract you” … then it's fine if it's like a fun, like childish name … Then, you would know that … it's still part of the … stopping smoking thing |
|  | P08: I agree [the game’s name should reference smoking] because if you have too many things that are evolving away from the reason that you're on the app, you might forget the reason that you're on the app … Names like that, it might take away from the fact that you're quitting smoking or that you're on this app to quit smoking … It might take the idea away from the whole thing. |
|  | P21: Maybe have [the game] be … an obvious section of the app where you can click on it instead of being … where you have to go to drop-down menu or … have pictures of the games as opposed to just the names of them … Things like that. Like, user friendly … kind of ideas. |
|  | P20: I think that there should be other games that should be available also as a choice. |
|  | P08: I like the game idea. I think maybe … it would be beneficial [if] … the game had something to do with … smoking rather than just some random game. |

Participant ID appears before each quote for attribution.

Total number of suggestion quotes = 33.
